# Low‐rank motion correction for accelerated free‐breathing first‐pass myocardial perfusion imaging

**DOI:** 10.1002/mrm.29626

**Published:** 2023-03-02

**Authors:** Gastao Cruz, Alina Hua, Camila Munoz, Tevfik Fehmi Ismail, Amedeo Chiribiri, René Michael Botnar, Claudia Prieto

**Affiliations:** ^1^ School of Biomedical Engineering and Imaging Sciences King's College London London UK; ^2^ Escuela de Ingeniería, Pontificia Universidad Católica de Chile Santiago Chile; ^3^ Millenium Institute for Intelligent Healthcare Engineering iHEALTH Santiago Chile

**Keywords:** accelerated scan, cardiac MR, free breathing, low rank, motion correction, myocardial perfusion

## Abstract

**Purpose:**

Develop a novel approach for accelerated 2D free‐breathing myocardial perfusion via low‐rank motion‐corrected (LRMC) reconstructions.

**Methods:**

Myocardial perfusion imaging requires high spatial and temporal resolution, despite scan time constraints. Here, we incorporate LRMC models into the reconstruction‐encoding operator, together with high‐dimensionality patch‐based regularization, to produce high quality, motion‐corrected myocardial perfusion series from free‐breathing acquisitions. The proposed framework estimates beat‐to‐beat nonrigid respiratory (and any other incidental) motion and the dynamic contrast subspace from the actual acquired data, which are then incorporated into the proposed LRMC reconstruction. LRMC was compared with iterative SENSitivity Encoding (SENSE) (itSENSE) and low‐rank plus sparse (LpS) reconstruction in 10 patients based on image‐quality scoring and ranking by two clinical expert readers.

**Results:**

LRMC achieved significantly improved results relative to itSENSE and LpS in terms of image sharpness, temporal coefficient of variation, and expert reader evaluation. Left ventricle image sharpness was approximately 75%, 79%, and 86% for itSENSE, LpS and LRMC, respectively, indicating improved image sharpness for the proposed approach. Corresponding temporal coefficient of variation results were 23%, 11% and 7%, demonstrating improved temporal fidelity of the perfusion signal with the proposed LRMC. Corresponding clinical expert reader scores (1–5, from poor to excellent image quality) were 3.3, 3.9 and 4.9, demonstrating improved image quality with the proposed LRMC, in agreement with the automated metrics.

**Conclusion:**

LRMC produces motion‐corrected myocardial perfusion in free‐breathing acquisitions with substantially improved image quality when compared with iterative SENSE and LpS reconstructions.

## Abstract 2

Click here for author‐reader discussions

**Abbreviations**
ADMM: AlternatingDirection Method of Multipliers
BLAST: Broad‐use Linear AcquisitionSpeed‐up Technique
CoV: Coefficient ofVariation
ESPIRiT: Eigenvalue Iterative Self‐consistent Parallel Imaging Reconstruction
FOCUSS: FOCal Underdetermined SystemSolver
FOV: Field of View
GRAPPA: Generalized Autocalibrating PartiallyParallel Acquisitions
HD‐PROST: High‐dimensional patch‐based reconstruction
ICA: Independent Component Anaylsis
itSENSE: iterativeSensitivity Encoding
LpS: Low Rank plusSparse
LRMC: Low Rank MotionCorrection
LV: Left Ventricle
MAE: Mean AbsoluteError
MRF: MagneticResonance Fingerprinting
PCA: Principal Component Analysis
RV: Right Ventricle
SENSE: Sensitivity Encoding
SLR: Sparse and Low Rank
SVD: Singular ValueDecomposition
TE: Echo Time
TR: Repetition Time
WET: Watersuppression Enhanced through T1 effects

## 1 INTRODUCTION

First‐pass myocardial MR perfusion imaging is a valuable MR application for diagnosing ischemia and coronary artery disease.^1^, ^2^ This technique aims to capture the “real‐time” perfusion in the myocardium immediately after injection of contrast agents, as they make their first pass through the circulatory system. Consequently, high temporal resolution is desired. Moreover, large coverage and high spatial resolution are also needed to properly detect perfusion abnormalities in the left ventricle (LV), resulting in a challenging acquisition process. To satisfy all these requirements, perfusion sequences usually acquire three (saturation‐prepared) images per heartbeat, where each image is in a different slice and cardiac phase. In‐plane resolutions are about 2 mm with acquisition windows up to 200 ms (per image) and a total scan time up to 60 s. In practice, data are acquired under partial breath‐hold, or completely free‐breathing. To improve spatial resolution, coverage and image quality, as well as to improve robustness to motion, several undersampled and motion‐corrected reconstruction–based approaches have been developed over the years.

Initial accelerated methods have modeled undersampling jointly in space and time, exploiting redundant information in the acquired data, leading to k‐t Broad‐use Linear Acquisition Speed‐up Technique/k‐t SENSitivity Encoding,^3^ k‐t Generalized Autocalibrating Partially Parallel Acquisition,^4^ and k‐t PCA^5^ (principal component analysis), among others. These frameworks have found great success in clinical practice,^6^, ^7^ although they are sensitive to respiratory motion due to inconsistencies between the training data and the acquired data. Respiratory gating is not useful in myocardial perfusion imaging due to the temporal resolution requirement; however, prospective slice‐tracking has been proposed for translational corrections.^8^ A variety of compressed sensing–based methods have been proposed for accelerated dynamic imaging, including k‐t SPARSE,^9^ k‐t SLR,^10^ k‐t FOCUSS,^11^ k‐t Group Sparse,^12^ k‐t PCA with motion correction,^13^ localized spatio‐temporal constraints,^14^ low‐rank plus sparse (LpS),^15^ and motion‐compensated independent component analysis (ICA).^16^ These approaches exploit the redundant information in dynamic contrast imaging (as the underlying geometry remains the same), via low‐rank structures and/or sparse domains. In most cases, motion causes an increase in the effective rank or reduced sparsity of the model, leading to a loss in performance. Some of these methods^10^, ^11^, ^16^ are better suited to dealing with motion, as they perform image‐based motion compensation, or exploit motion differences between frames. If motion is known, it can be incorporated into the reconstruction process. Fully elastic motion can be included into the regularization term of the reconstruction,^13^ improving the sparsity of the domain. Alternatively, it may be integrated into to the data‐consistency term, which may lead to improved performance.

Generalized motion‐corrected reconstructions have been proposed for MR^17^ and evaluated for different organs in multiple studies.^18^, ^19^, ^20^, ^21^, ^22^, ^23^ Nevertheless, the formalism developed in Batchelor et al.^17^ assumes that the image is acquired in a steady state (single contrast), which is not the case for several applications, including first pass myocardial perfusion imaging. In recent years, subspace‐constrained reconstructions have been explored, particularly in parametric mapping applications, in which dynamic contrast information is incorporated into the forward model.^24^, ^25^, ^26^, ^27^, ^28^, ^29^ However, these approaches assume that the imaging object remains in the same position as contrast evolves, which is also not guaranteed in myocardial perfusion scans. We have recently proposed a novel reconstruction method to perform generalized motion correction using a global low‐rank model (low‐rank motion correction [LRMC]),^30^ simultaneously leveraging redundant contrast information while correcting for arbitrary motion. Originally proposed for MR fingerprinting (MRF),^31^ LRMC was able to correct for respiratory and cardiac motion in liver and cardiac fingerprinting applications.

In this study, we evaluate the feasibility of LRMC for first‐pass myocardial perfusion, aiming to improve image quality while correcting for respiratory and other incidental sources of motion. The proposed framework derives the patient‐specific global low‐rank subspace and motion fields from the acquired data via auxiliary reconstructions, and then incorporates that information into the LRMC reconstruction. Additionally, LRMC uses patch‐based low‐rank tensor regularization (HD‐PROST)^32^ to enable high acceleration factors. The proposed approach was evaluated in 10 patients and compared with conventional iterative SENSE (itSENSE)^33^ and LpS^15^ reconstructions.

## 2 METHODS

### 2.1 Myocardial perfusion LRMC framework

**FIGURE 1:**
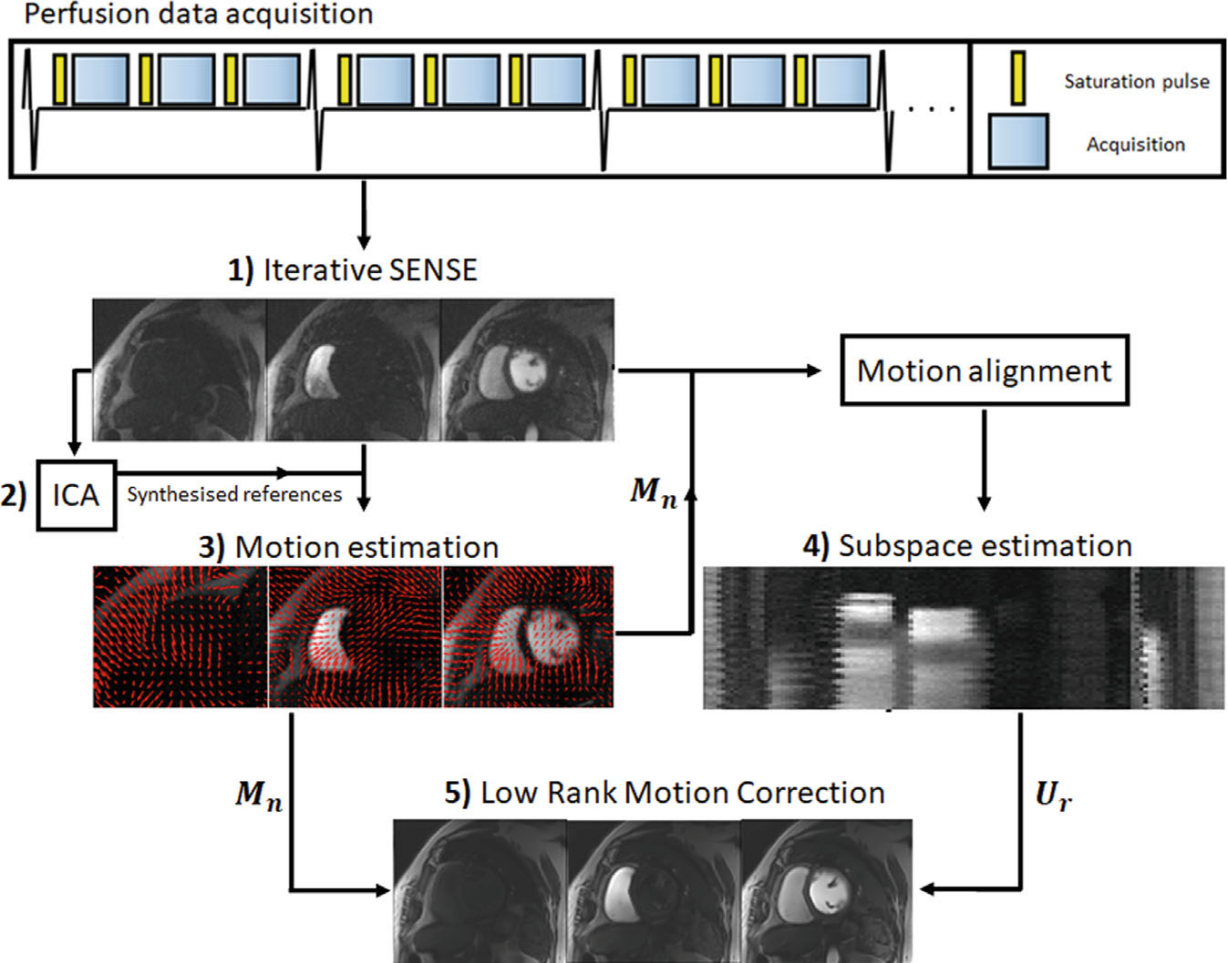
Diagram of the proposed framework reconstruction framework, divided into five steps: (1) Auxiliary iterative SENSE (itSENSE) reconstructions are performed to estimate motion and contrast subspace; (2) independent component analysis (ICA) is used to synthesize a set of images in fixed motion states to act as image‐registration references; (3) motion estimation is performed via image registration; (4) low‐rank subspace estimation is performed on the auxiliary itSENSE images after being motion‐aligned; and (5) the estimated motion and contrast subspace are used for the low‐rank motion correction (LRMC) reconstruction

The LRMC reconstruction requires knowledge of the patient motion and low‐rank subspace of the contrast dynamics. Both of these operators (along with coil sensitivities) are derived from the acquired data in the proposed framework, which is divided into five steps (Figure 1): (1) auxiliary itSENSE reconstruction, (2) ICA motion references, (3) motion estimation via image registration, (4) low‐rank subspace estimation, and (5) LRMC reconstruction. In Step 1, coil sensitivities are estimated from the acquired data via ESPIRiT^34^ and used to reconstruct the perfusion data using itSENSE. These auxiliary images are processed through an ICA pipeline to synthesize a perfusion image series with minimal motion, similar to what has been proposed previously.^16^ Briefly, Step 2 performs the following operations: (i) We compute the ICA(X)=WX, where X is the itSENSE reconstruction re‐arranged as a Casorati matrix and W is a matrix of transformation weights, using a preselected number of principal components; (ii) the independent component $W_{i}$ associated with motion (predominantly respiratory motion) is identified by finding the component with the largest frequency in the range of respiratory motion in the Fourier domain of W; (iii) a new motionless ICA basis $W^{\prime }$ is created, where the component $W_{i}=0$; and (iv) a set of motion‐free references are obtained via $X^{\prime }={\left(W_{i}\prime \right)}^{H}{W_{i}}^{\prime }X$. The image series $X^{\prime }$ should approximate the contrast evolution in X while suppressing most of the motion therein, resulting in a co‐registered perfusion series. Image registration with varying contrasts is a challenging problem. For that reason, we use $X^{\prime }$ as references for the image registration in Step 3, which provides the forward motion fields M at each time‐frame of the perfusion acquisition (i.e., for each heartbeat), using a B‐spline free‐form deformation motion model for registration.^35^, ^36^ In Step 4, the estimated motion is used to align the image series X into the reference motion state, followed by a singular value decomposition (SVD) of that series $\mathrm{SVD}\left(M^{H}X\right)=\mathrm{US}V^{H}$, where U, S, and V contain the left singular vectors, singular values, and right singular vectors of $M^{H}X$ (i.e., the motion‐aligned image series). The effective rank r of this matrix can be determined by finding the value that captures a predetermined value of the matrix energy,^24^ yielding the low‐rank subspace created by $U_{r}$, the truncation of U to rank r. Although $X^{\prime }$ could be used to extract the subspace, some contrast may be lost after ICA filtering; therefore, it is preferable to estimate it from the original (now motion‐aligned) data. Having the data co‐registered for the SVD step is also relevant, as some modes of variation may capture motion, which is not intended because the motion will be corrected during the LRMC reconstruction through M; thus, $U_{r}$ should only be capturing contrast variations. M and $U_{r}$ can be reshaped to apply the corresponding operations on vectorized images and used in the LRMC reconstruction,^30^ in Step 5:

(1) $$\begin{array}{ll} & \left(\hat{y},{\hat{T}}_{b}\right)=\underset{y,T_{b}}{\text{argmin}}\;\frac{1}{2}{\left\|\text{AFCM}U_{r}y-k\right\|}_{2}^{2}+\lambda \sum_{b}{\left\|T_{b}\right\|}_{*}\\ & \;s.t.T_{b}=Q_{b}\left(y\right) \end{array}$$

where A are the sampling matrices for all motion states; F is the Fourier transform; C are the coil sensitivities; k are the acquired data; and y are (motion‐corrected) *singular* (i.e., compressed) images. $U_{r}$ projects data from the compressed space into the temporal perfusion series, whereas $U_{r}^{H}$ projects data from the “beat‐to‐beat” temporal space back into the low‐rank subspace. According to the forward model $\text{AFCM}U_{r}$, y gets projected into motion‐corrupted k‐space in the temporal domain. The Hermitian transpose of the model corresponds to $U_{r}^{H}M^{H}C^{H}F^{H}A^{H}$. M in the forward model applies the motion from one reference motion state toward every other, corresponding to the MR acquisition. $M^{H}$ applies motion from all motion states toward the reference motion state, effectively motion‐correcting the perfusion series, which is then primed to be compressed via $U_{r}^{H}$ because the series is now co‐registered. HD‐PROST^32^ regularization is used, in which $Q_{b}$ generates a 3D tensor $T_{b}$ of voxels associated with the *b*th voxel by concatenating local voxels (within a local patch) along the first dimension, nonlocal voxels (from patches that exhibit structural similarity with the patch around *b*), and contrast voxels (along the compressed singular value domain). The formulation used in this study differs slightly from what was previously proposed in Cruz et al.^30^ specifically the commutation between F and $U_{r}$ (as $U_{r}$ can be reshaped such that it commutes with C and with M). Although previous studies in MRF^24^, ^26^ have produced similar results using both approaches, preliminary experiments with LRMC applied to perfusion data (now shown) indicated better contrast fidelity using the formulation in Eq. (1). Here, we solve the LRMC problem with ADMM (alternating direction method of multipliers).^37^

### 2.2 In vivo experiments

The proposed cardiac LRMC approach was evaluated in 10 patients with suspected cardiovascular disease with a 3T scanner (Achieva; Philips, Best, the Netherlands) using a 28‐channel cardiac coil. The study was approved by the institutional review board, and written informed consent was obtained from all subjects according to institutional guidelines. Acquisitions were performed during patient rest, using 0.15 mmol/kg of Gadovist. Patients were instructed to breathe normally. Main imaging parameters included three short‐axis slices, FOV = 256 × 256 mm^2^, 10‐mm slice thickness, resolution = 2 × 2 mm^2^, TE/TR = 1.6/3.5 ms, radial golden angle, flip angle = 15°, WET (water suppression enhanced through T_1_ effects) saturation pulse,^38^ 100‐ms saturation delay, and nominal scan time of approximately 60 s. Patients were instructed to breathe normally during the scan. A total of 53 radial lines (on average) were acquired per slice, per heartbeat, depending on the patient's heartrate. Data were reconstructed using conventional iterative SENSE,^33^ LpS,^15^ and the proposed LRMC.

### 2.3 Reconstruction parameters

ItSENSE used six iterations and was solved with the conjugate gradient algorithm. LpS used a low‐rank regularization $\lambda_{L}=0.1$ and a sparse regularization $\lambda_{s}=0.1$; six iterations were used in the method proposed in Otazo et al.^15^ For the proposed LRMC framework, ICA used five modes and automatically removed the ICA vector associated with the dominant frequency in the range of (0.2, 0.5) Hz; NiftyReg^36^ was used for image registration. ADMM used 10 iterations for the outer loop with three iterations for the inner (conjugate gradient) loop, and HD‐PROST regularization $\lambda =1\times 10^{-3}$. Subspace estimation for the LRMC was performed using a large region of interest (ROI) around the heart selected in a semi‐automated fashion: the user marks the septum, and a fixed rectangular ROI is created. The corresponding global low rank was selected to capture over 96% of the matrix energy (considering the sum of squared singular values), resulting in a rank of 8. All parameters were experimentally determined by performing a grid search in a set of reconstructions in two representative cases and choosing adequate parameters in terms of image quality and signal fidelity. Reconstructions were performed on a Linux workstation with 12 Intel Xeon X5675 (3.07 GHz) processors and 200 GB RAM. ItSENSE took approximately 10 min; LpS took approximately 10 min; and the proposed framework took approximately 70 min (with 10 min for initial itSENSE reconstruction, 20 min for nonrigid motion estimation, and 40 min for the final LRMC reconstruction), per slice.

### 2.4 Statistical analysis

Sharpness was measured in three key frames of the perfusion sequence (right ventricle [RV] enhancement [in the RV/myocardial boundary], LV enhancement [LV/myocardial boundary], and myocardial enhancement [LV/myocardial boundary]) for each subject and each slice. A total of 10 line profiles were manually drawn for each case and used to estimate $\text{sharpness}={\left|p_{80}-p_{20}\right|}^{-1}$, where $p_{80}$ ($p_{20}$) is the pixel location at 80% (20%) of the maximum intensity along the one‐dimensional line being evaluated.^39^, ^40^ The same set of (three) frames was used to evaluate each method. For each frame (and each reconstruction method), 10 lines were manually drawn to measure sharpness. Separate line drawings were required, as the three reconstructions under evaluation are not co‐registered. Aiming for an equal evaluation, lines were drawn carefully and aiming to be consistent between methods. The average sharpness over the 10 lines was averaged to produce each sharpness measurement. The fidelity of the perfusion curve was assessed by computing the coefficient of variation (CoV) relative to the median filtered curve, measuring residual aliasing and motion errors, similar to previous work.^13^ This analysis was performed for each myocardial segment of the bullseye plot,^41^ including three slices. Two expert clinical readers, with 12 and 2 years of experience in cardiac MR, respectively, were asked to assess the image quality of itSENSE, LpS, and the proposed LRMC. First, they were asked to score image quality according to the following scale: 5, Excellent image quality: no visible aliasing, blurring, or noise artifacts (no effect on diagnostic confidence); 4, Good image quality: minor aliasing, blurring, or noise artifacts (minimal effect on diagnostic confidence); 3, Acceptable image quality: some aliasing, blurring, or noise artifacts (minor loss of diagnostic confidence); 2, Mediocre image quality: considerable aliasing, blurring, or noise artifacts (considerable loss of diagnostic confidence); 1, Poor image quality: significant aliasing, blurring, or noise artifacts dominate (major loss of diagnostic confidence/nondiagnostic). Additionally, readers were asked to rank each reconstruction from 1 (worst) to 3 (best). Statistical significance for reader scores and rankings were assessed with the Wilcoxon signed‐rank test. Remaining metrics were assessed via two‐sample Student's t‐test, considering two‐tailed values of *p* < 0.05 as significant. Data and code will be made available by the authors upon reasonable request.

#### 2.4.1 Simulations

A numerical phantom, derived from a perfusion data set reconstructed with LRMC in this study, was used in simulations to assess the performance relative to a known ground truth. The CoV of the median signal and mean absolute errors (MAEs) were evaluated (after normalizing the image series to [0,1]). Additionally, LRMC was performed using varying degrees of motion errors, from 0% motion (i.e., no motion in the model, −100% error with respect to the ground truth) to 200% motion (i.e., 100% error in the motion model with respect to the ground truth). The simulation considered a single slice with the in vivo estimated motion as ground truth and included the corresponding undersampled radial trajectory, white Gaussian noise with amplitude equal to 2% of the maximum value in k‐space and known coil sensitivities. Simulated data were reconstructed via zero‐filling (including coil sensitivities) and with LRMC (without regularization, for fairer comparison).

## 3 RESULTS

### 3.1 In vivo experiments

**FIGURE 2:**
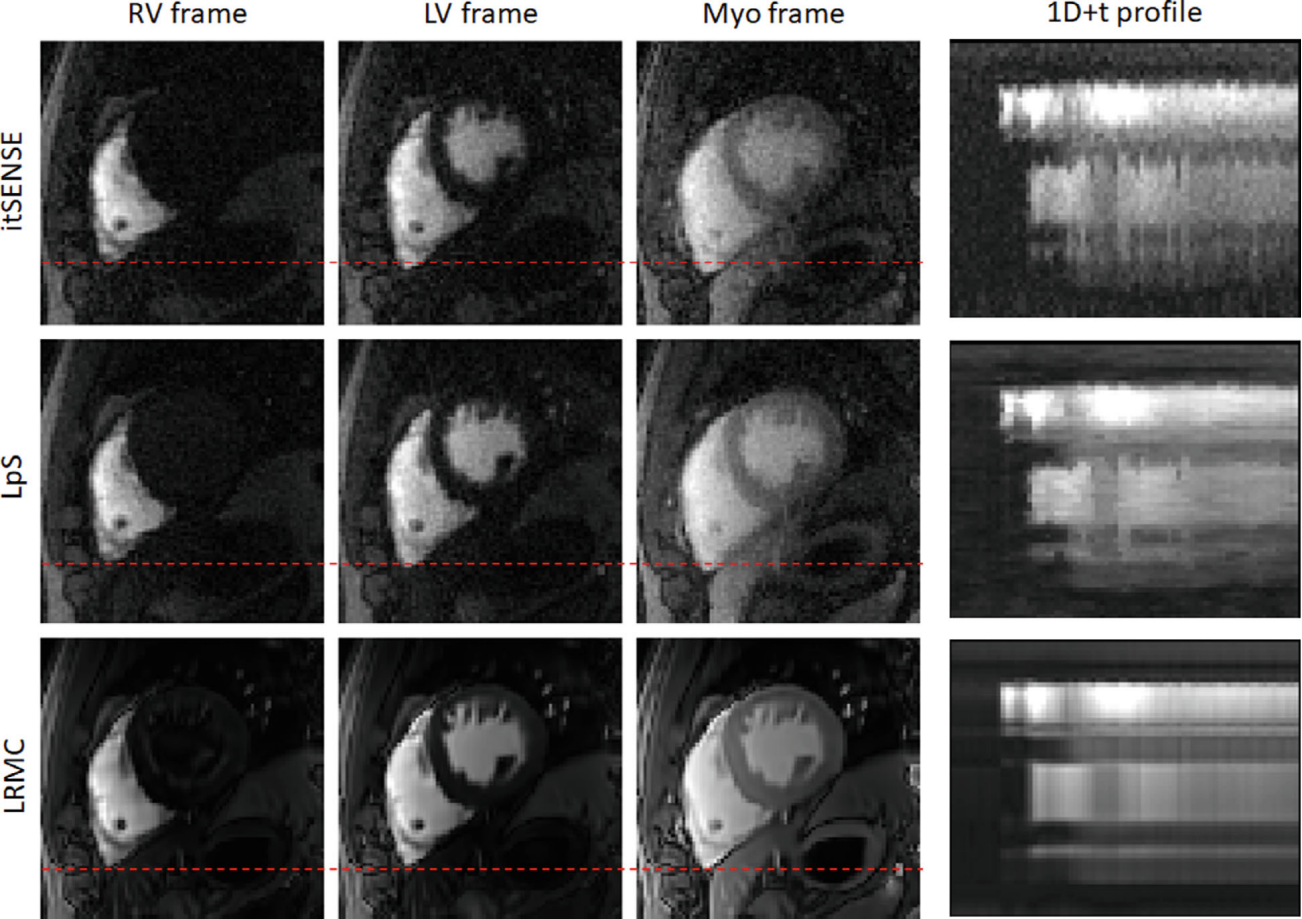
Perfusion frames corresponding to right ventricular (RV) enhancement, left ventricular (LV) enhancement, and myocardial (myo) enhancement, reconstructed with itSENSE, low‐rank plus Sparse (LpS), and the proposed LRMC for representative Patient A. Improved image quality, sharpness, and coregistration between frames is achieved with the proposed LRMC (the red dashed line facilitates comparison between frames in different motion states)

**FIGURE 3:**
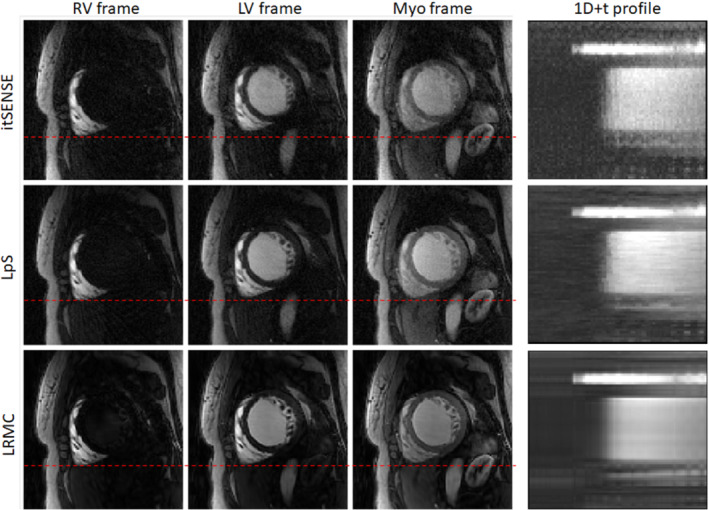
Perfusion frames corresponding to RV enhancement, LV enhancement, and myo enhancement, reconstructed with itSENSE, LpS, and the proposed LRMC for representative Patient B. Improved image quality, sharpness, and coregistration between frames is achieved with the proposed LRMC (the red dashed line facilitates comparison between frames in different motion states)

**FIGURE 4:**
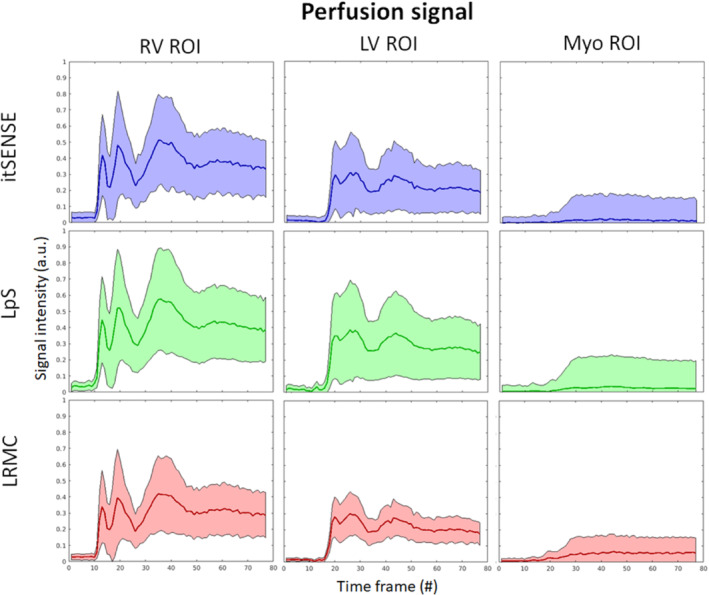
Contrast uptake for regions of interest (ROIs) in the RV enhancement, LV enhancement, and myo enhancement, reconstructed with itSENSE, LpS, and the proposed LRMC, for representative Patient A. The mean is represented by the line, whereas the variance is represented by the shaded area. Generally lower variance and less oscillations in the variance are observed with LRMC

**FIGURE 5:**
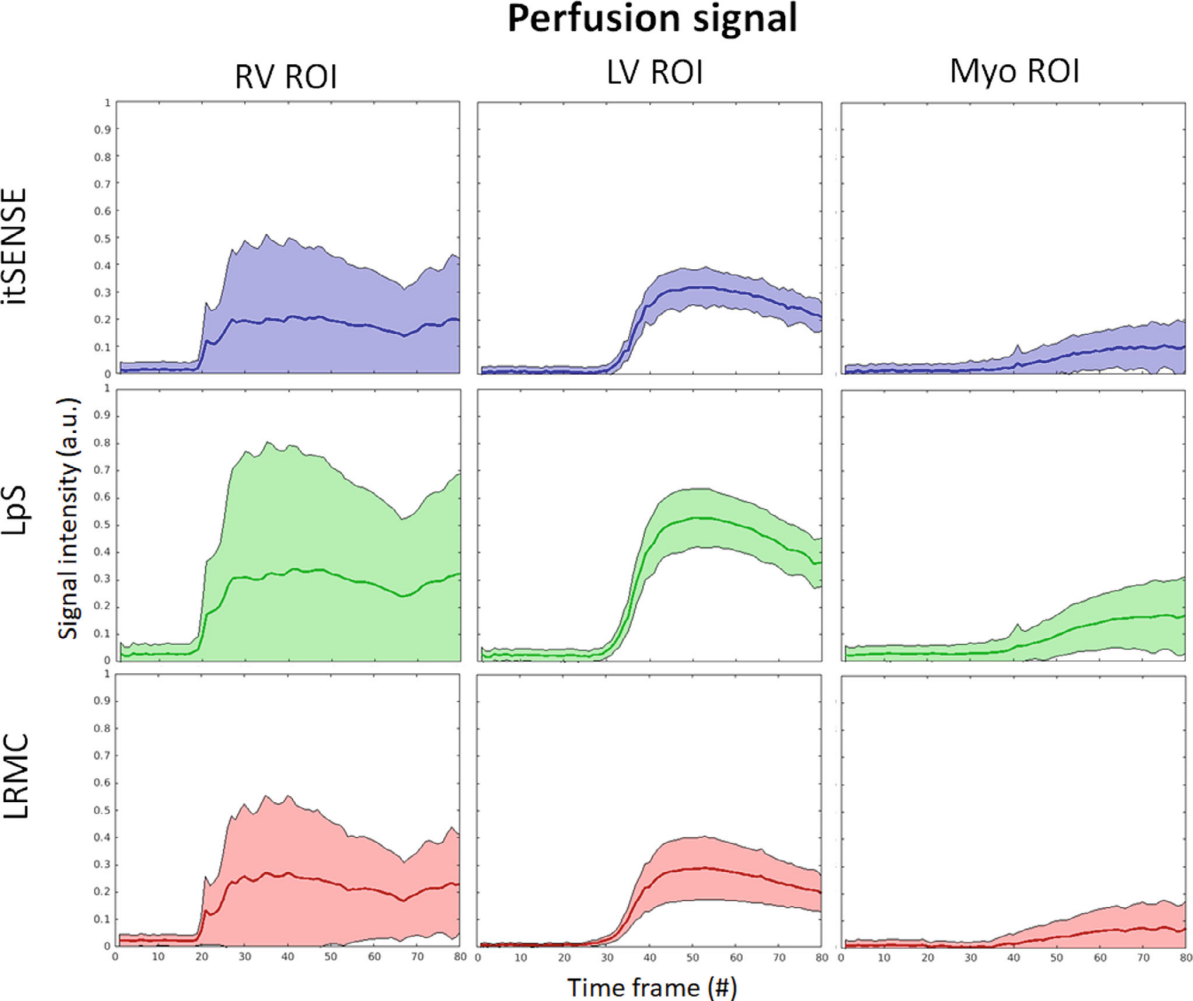
Contrast uptake for regions of interest in the RV enhancement, LV enhancement, and myo enhancement, reconstructed with itSENSE, LpS, and the proposed LRMC for representative Patient B. The mean is represented by the line, whereas the variance is represented by the shaded area. Generally lower variance and less oscillations in the variance are observed with LRMC

Key frames for RV, LV, and myocardial enhancement and one‐dimensional + t profiles are shown in Figures 2 and 3 for representative subjects A and B (red line is used to highlight the difference in motion states). Residual blurring, noise amplification, and streaking artifacts are present in the itSENSE reconstruction. Noise and streaking are reduced using LpS; further artifact suppression and increased image quality are obtained with the proposed LRMC. Furthermore, it can be observed that, although the motion states of itSENSE and LpS frames vary in time, LRMC are motion‐corrected to a single state. The performance of itSENSE, LpS, and LRMC in terms of image quality and motion correction can also be appreciated in Videos S1 and S2 for representative Subjects C and D. LRMC achieves higher image quality than itSENSE and LpS reconstructions; moreover, all frames are motion‐corrected to the same reference motion state. Because generalized motion correction is performed on a beat‐to‐beat fashion (i.e., no cardiac/respiratory binning), LRMC can correct for any type of motion that occurs during the scan: respiratory, cardiac, or residual bulk motion. This correction contributes to an improved characterization of the contrast uptake, as visualized in Figures 4 and 5 for the same subjects. All reconstruction methods correctly describe the contrast dynamics in the RV, LV, and myocardium; however, the LRMC generally presents smaller variances within each ROI throughout the perfusion series. Additionally, the variance itself presents fewer oscillations (usually associated with tissue boundaries) with LRMC. These reduced variations are partially due to the improved image quality, but primarily due to the coregistration of the series that happens after motion correction. To further evaluate the temporal fidelity of the perfusion series, the itSENSE and LpS reconstructions were motion‐aligned using the same motion fields in LRMC, and the intensity evolution was assessed for each myocardial segment. These temporal evolutions are plotted in Figures S5–S14 for each patient. Therein, we can observe that all three methods depict very similar contrast uptakes. LpS admits some temporal blurring (due to leveraging low‐rank properties without motion correction), which may bias the motion states at each frame, relative to itSENSE, from which the motion was estimated. This causes some of the residual oscillations in the signal for LpS seen in Figures S5–S14.

**FIGURE 6:**
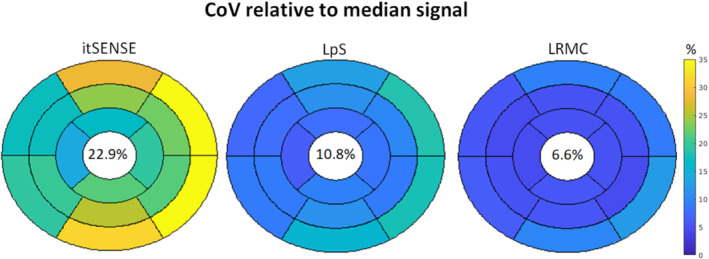
“Bullseye” plot for the coefficient of variation (CoV) of the temporal signal relative to the median filtered signal, reconstructed with itSENSE, LpS, and the proposed LRMC, averaged over the patient cohort. Generally higher variations are observed in the basal and lateral segments, with the lowest variations being achieved by the proposed LRMC

**FIGURE 7:**
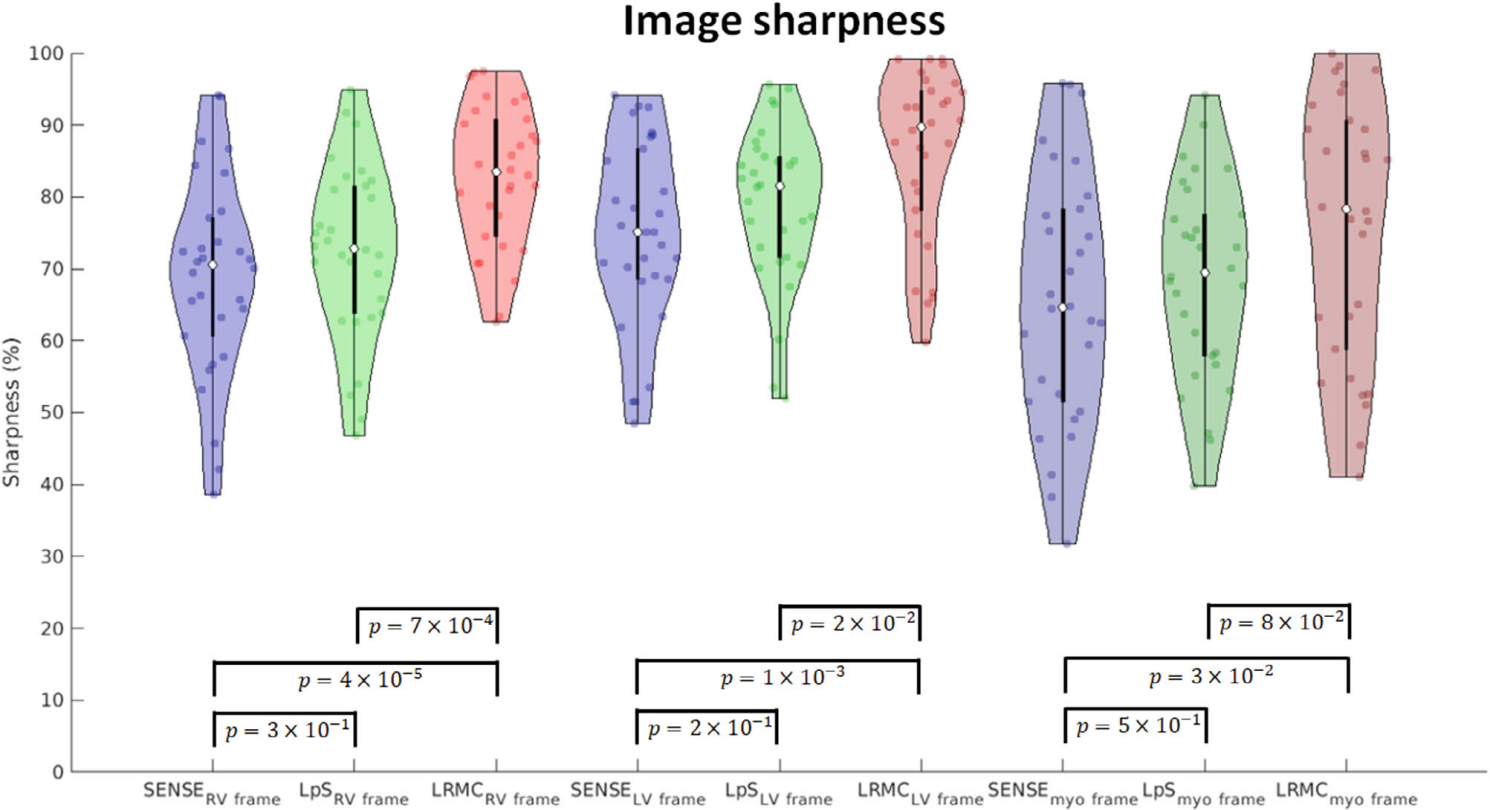
Violin plots of image sharpness measured in frames of RV enhancement, LV enhancement, and myo enhancement, reconstructed with itSENSE, LpS, and the proposed LRMC. Higher sharpness and more compact distributions were generally achieved by the LRMC

**FIGURE 8:**
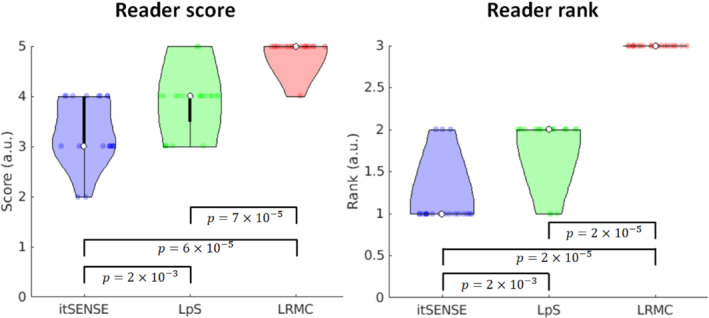
Violin plots of reader scores (in a range of 1 to 5) and reader rankings^1^, ^2^, ^3^ for itSENSE, LpS, and the proposed LRMC. LRMC consistently achieved the highest scores and rankings

The temporal fidelity of the series was also evaluated in a quantitative fashion via the CoV relative to the median perfusion signal, similar to previous work.^13^ For all reconstruction methods, higher CoVs were generally observed in basal and/or lateral segments, using the conventional “bullseye” segmental analysis (Figure 6).^41^ The mean CoV over the heart was approximately 23% for itSENSE, 11% for LpS, and 7% for LRMC. Both LpS and LRMC were significantly different from itSENSE; moreover, LRMC was significantly different from LpS. Image sharpness was evaluated for the whole cohort in key frames of RV enhancement, LV enhancement, and myocardial enhancement (Figure 7). RV sharpness was approximately 69% for itSENSE, 73% for LpS, and 83% for LRMC; LV sharpness was approximately 75% for itSENSE, 79% for LpS, and 86% for LRMC; myocardial sharpness was approximately 66% for itSENSE, 69% for LpS, and 76% for LRMC. No significant differences were observed between itSENSE and LpS. LRMC sharpness was significantly higher than itSENSE in all cases; it was also significantly higher than LpS for the RV and LV frames. Finally, two readers provided image scores and rankings of the three reconstruction methods (Figure 8). The itSENSE, LpS, and LRMC methods achieved mean scores of 3.3, 3.9 and 4.9, respectively; significant differences were observed between itSENSE and LpS, between itSENSE and LRMC, and between LpS and LRMC. The itSENSE, LpS, and LRMCS methods had a mean rank of 1.1, 1.9 and 3.0, respectively; again, significant differences were observed between itSENSE and LpS, between itSENSE and LRMC, and between LpS and LRMC. Notably, LRMC achieved the maximum score in every case except for one; additionally, LRMC was ranked the best in every case. No pathological findings were present in the patient cohort. Peak respiratory diaphragmatic motion amplitudes were [11.6, 26.4, 18.0] mm ([min, max, mean]). Quantitative results for all metrics are compiled in Table 1.

**TABLE 1:** Summary of metrics evaluated to compare itSENSE, LpS, and the proposed LRMC. Image sharpness was evaluated in key frames of RV enhancement, LV enhancement, and myo enhancement. Temporal CoV was measured relative to the median signal. Reader image‐quality scores ranged from 1 to 5

	Sharpness RV (%)	Sharpness LV (%)	Sharpness myo (%)	Myo signal CoV (%)	Scores (a.u.)	Rank (a.u.)
itSENSE	68.8 ± 14.0	74.8 ± 13.0	65.8 ± 17.5	23.0 ± 8.3	3.3 ± 0.7	1.1 ± 0.4
LpS	72.5 ± 12.1	78.9 ± 11.1	68.6 ± 13.7	10.8 ± 5.5^a^	3.9 ± 0.6^a^	1.9 ± 0.4^a^
LRMC	82.8 ± 9.9^a^ ^,^ ^b^	85.9 ± 11.8^a^ ^,^ ^b^	75.5 ± 18.9^a^	6.5 ± 3.6^a^ ^,^ ^b^	4.9 ± 0.2^a^ ^,^ ^b^	3.0 ± 0.0^a^ ^,^ ^b^

^a^ Denotes significant difference to itSENSE.

^b^ Denotes significant difference between LpS and LRMC.

#### 3.1.1 Simulations

Representative frames of reconstructed images for simulation are shown in Figure S1. Similar to in vivo results, aliasing and noise amplification is considerably suppressed with LRMC. Evaluation of CoV relative to the median and MAE (Figure S2) demonstrated a general agreement between CoV and MAE measurements, being substantially lower for LRMC. Furthermore, CoV measurements were also in general agreement with in vivo results. Errors in the motion model can lead to artifacts in the resulting reconstructions, as demonstrated in Figure S3. Considerable blurring is observed for 0% motion, and noise amplification for 200% motion. In addition to blurring and noise amplification, some structured motion artifacts may occur due to errors in the motion model. In both cases, the frames are no longer perfectly coregistered. Corresponding American Heart Association (AHA) maps for the CoV of the median and MAE are shown in Figure S4. The CoVs are significantly lower than zero‐filling, even from extreme cases of 0% or 200% motion. This is because varying contrast data are shared within the low‐rank model, leading to an averaging of motion states even if perfect coregistration is not achieved. Nevertheless, the minimum CoV is achieved for the 100% motion (i.e., ground‐truth motion). We can also observe a correspondence of the CoV to the MAE, resulting in the lowest errors for 100% motion and increasing for higher errors in motion. Additionally, higher errors were observed for large positive errors in motion (i.e., 200% motion) rather than negative motion errors (or even absence of motion incorporated into the model).

## 4 DISCUSSION

In this work, we evaluated the feasibility of a novel reconstruction for free‐breathing, first‐pass myocardial perfusion. The proposed LRMC combines subspace‐constrained models with a generalized motion‐corrected forward model to improve image quality, temporal fidelity, and coregistration of the perfusion series. Moreover, high‐dimensional patch‐based regularization (HD‐PROST) is used to enable high acceleration factors. Previous methods for myocardial perfusion have leveraged the low‐rank properties of the series or attempted to incorporate patient motion into the reconstruction process. LRMC corrects for arbitrary motion in a patient‐specific subspace, making it an efficient method to exploit redundant temporal information and correct for motion simultaneously.

LRMC consistently outperformed itSENSE and LpS in all the metrics evaluated: image sharpness, temporal fidelity of the signal, expert reader scoring, and reader ranking. Undersampled radial iterative SENSE will admit residual aliasing in the reconstructions, partially in the form of blurring. LpS will have less residual aliasing, although will incur in some blurring artifacts when the model fails to fully resolve the motion in the series. LRMC is better posed than iterative SENSE (due to the subspace in the forward model) and does not suffer from motion blurring problems (due to the motion fields in the forward model), leading to improved sharpness relative to the other approaches. Residual “flickering” artifacts were also observed in some data sets across all three reconstructions (e.g., temporal profiles in Figure 2), which may be related to errors in the saturation preparation.

The increase in apparent SNR in the LRMC is due to two reasons: (1) The subspace **Ur** in the forward model uses all the acquired data to reconstruct each perfusion frame (which is only possible because motion correction is also incorporated into the forward model; otherwise motion artifacts would arise); and (2) the patch‐based regularization HD‐PROST^1^ is used to suppress noise amplification and residual aliasing, similar to other forms of compressed sensing. Residual streaking, noise amplification, and blurring were present in the itSENSE reconstruction. Although streaking and noise were reduced using LpS, no significant improvements in sharpness were observed. This is due to some blurring induced by the LpS, as it attempts to leverage temporally redundant information in data with considerable respiratory motion. The LpS did produce some temporal smoothing, resulting in improved temporal CoV relative to itSENSE. Expert readers also found LpS to be significantly better than itSENSE. LRMC was significantly better than itSENSE in every metric evaluated; it was also significantly better than LpS in every metric except myocardial sharpness. The global low‐rank model of the LRMC provides considerable suppression of residual aliasing and other artifacts, which is enabled by the motion‐correction component. Moreover, the high‐dimensionality patch‐based HD‐PROST^32^ enables high‐quality reconstructions from highly undersampled or noisy data. In the absence of motion correction, this model would reduce to a low‐rank inversion reconstruction^24^, ^25^, ^26^ and likely produce motion artifacts (primarily in the form of blurring), as shown previously.^42^ This type of behavior was observed in the LpS reconstruction, leading to similar image sharpness as itSENSE, in contrast to improvements in signal CoV and reader evaluations.

The proposed framework performs beat‐to‐beat nonrigid motion correction, requiring estimation of patient‐specific motion fields. In this work, motion is estimated via image registration, which was successfully performed in the patient cohort evaluated. Because motion is estimated from a varying contrast series, a reference motion state with matching varying contrast was synthesized to facilitate the elastic registration process. In this initial framework, motion was estimated from a preliminary iterative SENSE reconstruction, susceptible to residual aliasing and noise amplification. These artifacts could affect the accuracy of the estimated motion and in turn propagate errors into the reconstructed image series. The challenges associated with motion estimation are one of the key limitations of the current framework, as errors in the motion model (in addition to coil sensitivities and contrast subspace) could propagate as coherent artifacts in the final reconstructed perfusion series. Simulations indicated that underestimation of the motion is likely to create blurring artifacts, whereas overestimating motion leads primarily to noise amplification. However, more structured artifacts may occur, which would be more detrimental to the diagnostic value of the images. This limitation could be reduced by using stronger preliminary reconstructions or more advanced approaches for motion estimation. We note that the simulations performed here are limited by the fact that the ground‐truth numerical phantom was obtained from a low‐rank reconstruction itself. Additional errors in motion, noise, and undersampling were considered for the simulation to help reduce this limitation of the simulation.

The patient cohort was instructed to breathe normally during the acquisition. The acquired data included mostly respiratory motion, but also cardiac motion in some cases (due to varying heartrates or potentially mistriggering), and rarely residual bulk motion. Because the proposed approach does not perform any “binning” along a prescribed motion dimension (e.g., respiratory dimension, cardiac dimension), it will correct for any type of in‐plane motion that occurs between heartbeats. However, no motion correction is performed within each cardiac acquisition window. This could lead to unresolved cardiac motion, which may cause dark rim artifacts^43^ in addition to conventional motion artifacts (primarily blurring in the case of the radial trajectory). Intra‐shot motion could in theory be corrected with the LRMC reconstruction; however, additional improvements to the framework would be needed to estimate the residual (primarily cardiac) motion within each shot. The acquisition used is 2D multislice, and the reconstruction is performed slice by slice. As such, the proposed framework is susceptible to through‐plane motion. Some through‐plane motion was observed in the diaphragm, typically due to deep breaths performed by the patient. Although we did not observe through‐plane or partial‐volume artifacts around the heart, these effects could happen, particularly in cases of deep breaths, unusual anatomy, or inaccurate short‐axis planning.

The proposed framework also requires an estimate of the temporal subspace, which can be estimated from k‐space data, low‐resolution training images, or simulated signal models.^24^, ^44^, ^45^ Here, the subspace is estimated from auxiliary itSENSE images. While we do not expect residual noise or incoherent aliasing to affect subspace estimation, other confounders like in‐plane and through‐plane motion may alter the subspace. For this reason, estimation of the contrast subspace is performed in a motion‐aligned perfusion series. The choice of rank for subspace‐constrained reconstructions (here, LpS and LRMC) is a key parameter that determines a trade‐off between accuracy and precision. A relatively low rank is required to suppress noise and incoherent aliasing; however, it may also produce artificial contrast in noisy frames or regions that do not contribute significantly to the main modes of variation in the perfusion series (which fundamentally determine the subspace). Temporal fidelity of the low‐rank‐based methods (LpS and LRMC) in the myocardium was in agreement with conventional iterative SENSE (as seen in Figures S5–S14). However, some contrast differences in the initial low‐signal/high‐noise frames could be observed (e.g., Videos S1 and S2). In this work, the choice of rank was fixed for all patients; however, future work will consider automated rank to ensure subspaces are optimal for each case.

The proposed LRMC reconstruction is computationally demanding, as the computational time increases with the number of motion states. The computational bottleneck is the LRMC reconstruction itself, with the auxiliary reconstruction and motion‐estimation processes requiring less computations. In the context of this framework, each heartbeat corresponds to a motion state; therefore, the reconstruction time is approximately proportional to the acquisition time, and in turn by the number of non‐uniform fast Fourier transforms required. In addition to implementation in high‐performance languages (e.g., C++) and using GPU‐based nonuniform fast Fourier transforms,^46^, ^47^ future work will consider a reformulation of this problem in the context of deep learning to improve motion estimation and reconstruction speeds, while potentially further increasing performance, as demonstrated in recent studies.^48^, ^49^, ^50^

Recent methods like Multitasking^45^ resolve the motion instead of correcting for it, generating a set of contrast‐resolved images in all the motion states (e.g., respiratory, cardiac) considered. Although contrast information is incorporated into the reconstruction in a somewhat similar fashion, LRMC and Multitasking differ substantially on the motion component. LRMC requires estimation of dense motion fields that are incorporated in a low‐rank matrix problem, whereas Multitasking requires the estimation of the motion subspace, which is in turn incorporated into a low‐rank tensor problem. In this study, we evaluated the feasibility of LRMC and compared it with established methods such as itSENSE and LpS. Future work will consider comparisons with additional methods proposed for myocardial perfusion.

One of the main limitations in this study is that LRMC was only evaluated in a relatively small patient cohort with no pathological findings, using only rest scans. As such, further patient experiments will be needed to determine the added value of LRMC in detecting perfusion deficits or other markers. We hypothesize that LRMC could enable and improve other applications for first‐pass perfusion. LRMC could be used to simultaneously reconstruct rest and stress perfusion data, improving image quality and motion‐correcting the full image series for facilitated quantitative perfusion analysis and the automated computation of myocardial perfusion reserve. Moreover, if additional data are acquired in the same geometry (e.g., Late Gadolinium Enhancement), that data could also be considered for joint reconstruction, improving reconstruction quality for all data sets. Although shown here for 2D imaging, LRMC may also be applied for 3D perfusion,^51^ similar to previously demonstrated 3D MRF, to improve performance or enable higher resolution/coverage. The current framework used a triggered acquisition and beat‐to‐beat motion correction. The LRMC could be applied to a “free‐running” acquisition, similar to previous studies with Multitasking^45^; however, further investigation is needed. Cardiac motion could be estimated for each cardiac phase (in addition to respiratory motion), potentially requiring binning strategies for motion estimation (depending on the acceleration factor). However, such a sequence may improve the effective temporal resolution from the order of one heartbeat to the order of the TR, allowing for a much better temporal characterization of the perfusion dynamics and temporally resolved parametric mapping.^45^ Because the computational burden of the LRMC increases with the number of motion states, reconstruction times could become prohibitively long, especially for 3D. Corresponding deep‐learning solutions with very fast inference times may help reduce the computational burden. These approaches could facilitate the development of multi‐parametric quantitative perfusion for improved diagnosis.

## 5 CONCLUSION

A novel LRMC reconstruction is used to enable free‐breathing, first‐pass myocardial perfusion imaging with superior image quality than state‐of‐the‐art reconstruction techniques. The proposed LRMC demonstrates significant improvements in comparison to iterative SENSE and LpS in terms of sharpness, temporal fidelity, and expert clinical evaluation.

## Supporting information


**Video S1.** Animated perfusion series for three slices reconstructed with iterative SENSE (itSENSE, left column), low‐rank plus sparse (LpS, middle column), and the proposed low‐rank motion correction (LRMC, right column) for representative Subject C. Residual artifacts and motion between frames are apparent in itSENSE and LpS; however, those are substantially reduced with the proposed LRMC

**Video S2.** Animated perfusion series for three slices reconstructed with itSENSE (left column), LpS (middle column), and the proposed LRMC (right column) for representative Subject D. Residual artifacts and motion between frames are apparent in itSENSE and LpS; however, those are substantially reduced with the proposed LRMC

**Figure S1.** Representative frames of a simulated perfusion acquisition reconstructed with zero‐filling (with coil maps), low‐rank motion correction (LRMC; non‐regularized), and the ground truth. Image quality is in general agreement with results observed in vivo
**Figure S2.** Coefficient of variation as a surrogate to capture temporal fidelity and mean absolute error (MAE) measured in simulated numerical data. For both metrics, larger errors are obtained for zero‐filled reconstructions (including coil maps) than for LRMC (without regularization)
**Figure S3.** Representative frames from a simulation reconstructed with (unregularized) LRMC using varying degrees of motion errors. If motion is not incorporated into the model (0% motion), then the reconstruction produces primarily blurring artifacts. For extreme errors in the motion (200% motion [i.e., 100% error with respect to ground truth]), we observe primarily noise amplification
**Figure S4.** Coefficient of variation (CoV) and MAE for (unregularized) LRMC reconstructions considering a range of motion errors, from 0% (no motion) to 200% (i.e., 100% error with respect to ground truth). (B) CoVs increase with motion errors (i.e., away from 100% motion), although all cases are substantially lower than zero‐filing. MAEs present similar behavior, although higher errors are observed for large positive motion errors (200% [i.e., 100% error with respect to ground truth])
**Figure S5.** Signal evolutions of the perfusion series for Subject A, along each of the 16 American Heart Association (AHA) myocardial segments, for iterative SENSE (blue), low‐rank plus sparse (LpS, green), and the proposed LRMC (red). itSENSE and LpS have been motion‐aligned using the same motion fields in LRMC to facilitate the comparison of the perfusion temporal evolutions. All three methods present similar temporal evolutions
**Figure S6.** Signal evolutions of the perfusion series for Subject B, along each of the 16 AHA myocardial segments, for iterative SENSE (blue), LpS (green), and the proposed LRMC (red). itSENSE and LpS have been motion‐aligned using the same motion fields in LRMC to facilitate the comparison of the perfusion temporal evolutions. All three methods present similar temporal evolutions
**Figure S7.** Signal evolutions of the perfusion series for Subject C, along each of the 16 AHA myocardial segments, for iterative SENSE (blue), LpS (green), and the proposed LRMC (red). itSENSE and LpS have been motion‐aligned using the same motion fields in LRMC to facilitate the comparison of the perfusion temporal evolutions. All three methods present similar temporal evolutions
**Figure S8.** Signal evolutions of the perfusion series for Subject D, along each of the 16 AHA myocardial segments, for iterative SENSE (blue), LpS (green), and the proposed LRMC (red). itSENSE and LpS have been motion‐aligned using the same motion fields in LRMC to facilitate the comparison of the perfusion temporal evolutions. All three methods present similar temporal evolutions
**Figure S9.** Signal evolutions of the perfusion series for Subject E, along each of the 16 AHA myocardial segments, for iterative SENSE (blue), LpS (green), and the proposed LRMC (red). itSENSE and LpS have been motion‐aligned using the same motion fields in LRMC to facilitate the comparison of the perfusion temporal evolutions. All three methods present similar temporal evolutions
**Figure S10.** Signal evolutions of the perfusion series for Subject F, along each of the 16 AHA myocardial segments, for iterative SENSE (blue), LpS (green), and the proposed LRMC (red). itSENSE and LpS have been motion‐aligned using the same motion fields in LRMC to facilitate the comparison of the perfusion temporal evolutions. All three methods present similar temporal evolutions
**Figure S11.** Signal evolutions of the perfusion series for Subject G, along each of the 16 AHA myocardial segments, for iterative SENSE (blue), LpS (green), and the proposed LRMC (red). itSENSE and LpS have been motion‐aligned using the same motion fields in LRMC to facilitate the comparison of the perfusion temporal evolutions. All three methods present similar temporal evolutions
**Figure S12.** Signal evolutions of the perfusion series for Subject H, along each of the 16 AHA myocardial segments, for iterative SENSE (blue), LpS (green), and the proposed LRMC (red). itSENSE and LpS have been motion‐aligned using the same motion fields in LRMC to facilitate the comparison of the perfusion temporal evolutions. All three methods present similar temporal evolutions
**Figure S13.** Signal evolutions of the perfusion series for Subject I, along each of the 16 AHA myocardial segments, for iterative SENSE (blue), LpS (green), and the proposed LRMC (red). itSENSE and LpS have been motion‐aligned using the same motion fields in LRMC to facilitate the comparison of the perfusion temporal evolutions. All three methods present similar temporal evolutions
**Figure S14.** Signal evolutions of the perfusion series for Subject J, along each of the 16 AHA myocardial segments, for iterative SENSE (blue), LpS (green), and the proposed LRMC (red). itSENSE and LpS have been motion‐aligned using the same motion fields in LRMC to facilitate the comparison of the perfusion temporal evolutions. All three methods present similar temporal evolutions
