# Supplementary material for: Low‐rank motion correction for accelerated free‐breathing first‐pass myocardial perfusion imaging
Source: Magn Reson Med. 2023 Mar 2;90(1):64–78. doi: 10.1002/mrm.29626 (PMC10952238; doi:10.1002/mrm.29626)
Supplement: Supplementary file 3 — Figure S1. Representative frames of a simulated perfusion acquisition reconstructed with zero‐filling (with coil maps), low‐rank motion correction (LRMC; non‐regularized), and the ground truth. Image quality is in general agreement with results observed in vivo Figure S2. Coefficient of variation as a surrogate to capture temporal fidelity and mean absolute error (MAE) measured in simulated numerical data. For both metrics, larger errors are obtained for zero‐filled reconstructions (including coil maps) than for LRMC (without regularization) Figure S3. Representative frames from a simulation reconstructed with (unregularized) LRMC using varying degrees of motion errors. If motion is not incorporated into the model (0% motion), then the reconstruction produces primarily blurring artifacts. For extreme errors in the motion (200% motion [i.e., 100% error with respect to ground truth]), we observe primarily noise amplification Figure S4. Coefficient of variation (CoV) and MAE for (unregularized) LRMC reconstructions considering a range of motion errors, from 0% (no motion) to 200% (i.e., 100% error with respect to ground truth). (B) CoVs increase with motion errors (i.e., away from 100% motion), although all cases are substantially lower than zero‐filing. MAEs present similar behavior, although higher errors are observed for large positive motion errors (200% [i.e., 100% error with respect to ground truth]) Figure S5. Signal evolutions of the perfusion series for Subject A, along each of the 16 American Heart Association (AHA) myocardial segments, for iterative SENSE (blue), low‐rank plus sparse (LpS, green), and the proposed LRMC (red). itSENSE and LpS have been motion‐aligned using the same motion fields in LRMC to facilitate the comparison of the perfusion temporal evolutions. All three methods present similar temporal evolutions Figure S6. Signal evolutions of the perfusion series for Subject B, along each of the 16 AHA myocardial segments, for iterative SENSE (blue [file MRM-90-64-s002.docx]

Supporting Information Figures:


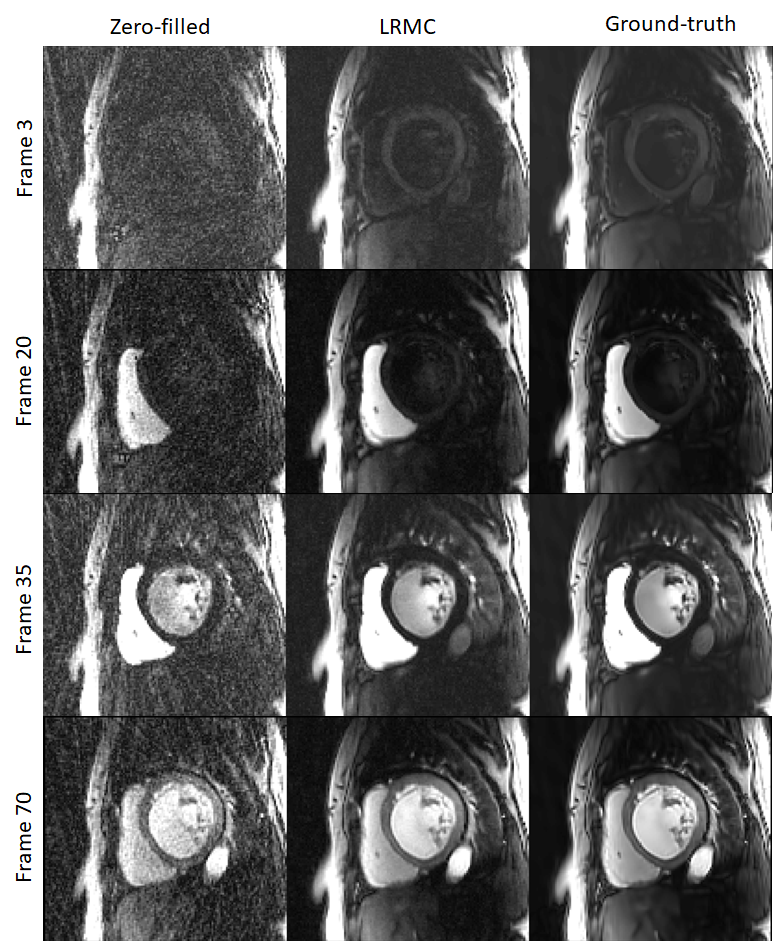


**Supporting Information Figure S1.** Representative frames of a simulated perfusion acquisition reconstructed with zero-filling (with coil maps), LRMC (non-regularized) and the ground-truth. Image quality is in general agreement with results observed in-vivo.


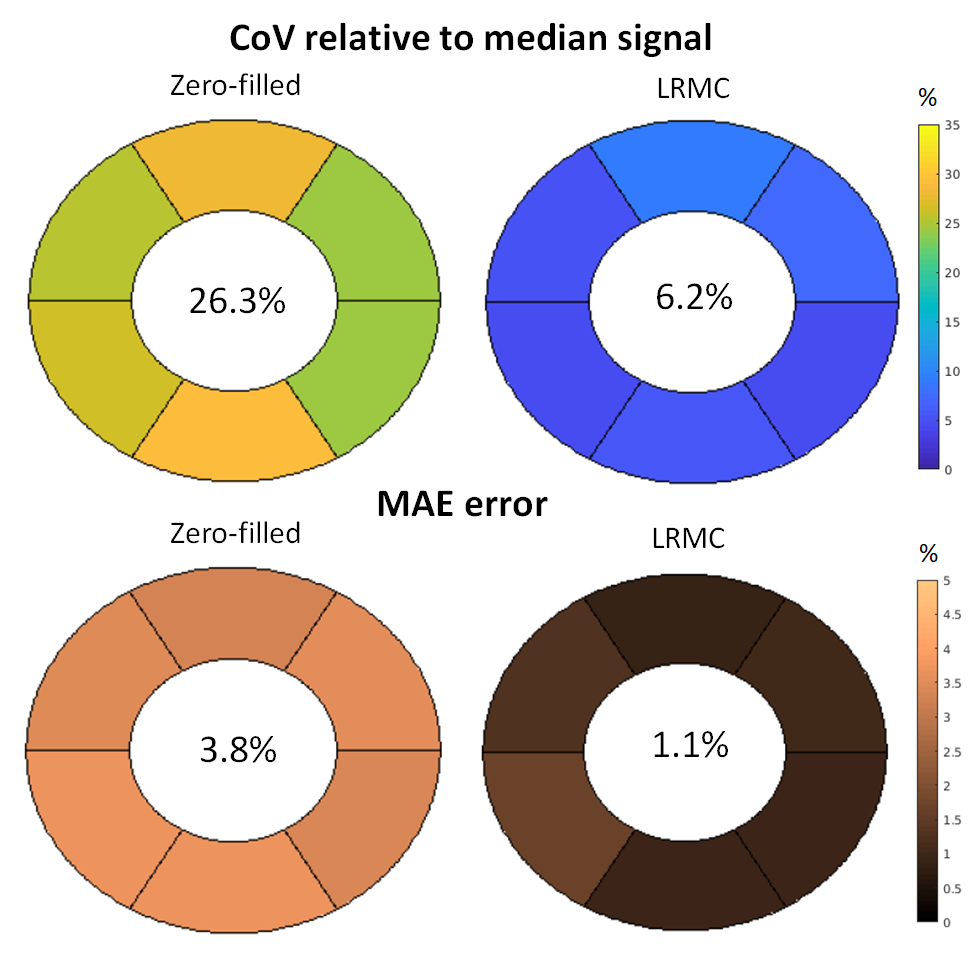


**Supporting Information Figure S2.** Coefficient of variation as a surrogate to capture temporal fidelity and mean absolute error (MAE) measured in simulated numerical data. For both metrics, larger errors are obtained for zero-filled reconstructions (including coil maps) than for LRMC (without regularization).


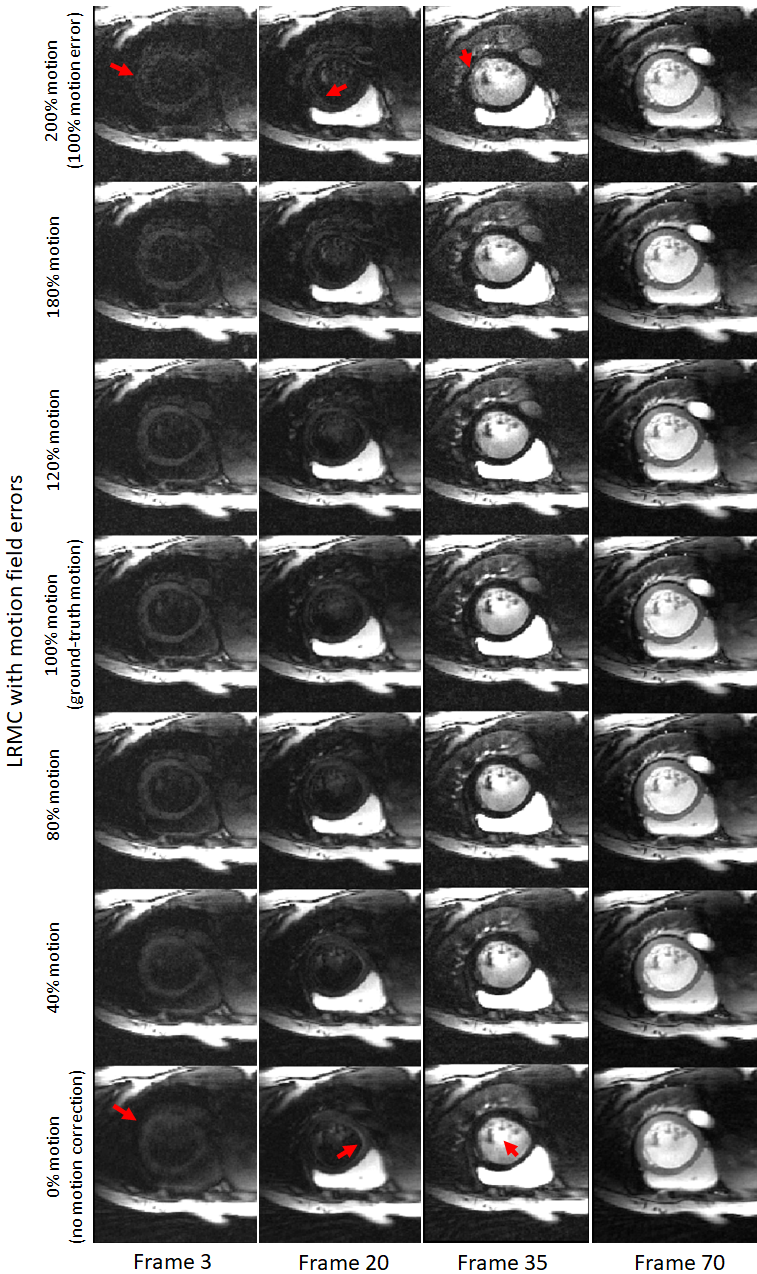


**Supporting Information Figure S3.**  Representative frames from a simulation reconstructed with (unregularized) LRMC using varying degrees of motion errors. If motion is not incorporated into the model (0% motion) then the reconstruction produces primarily blurring artefacts. For extreme errors in the motion (200% motion, i.e. 100% error with respect to ground-truth) we observe primarily noise amplification.

^
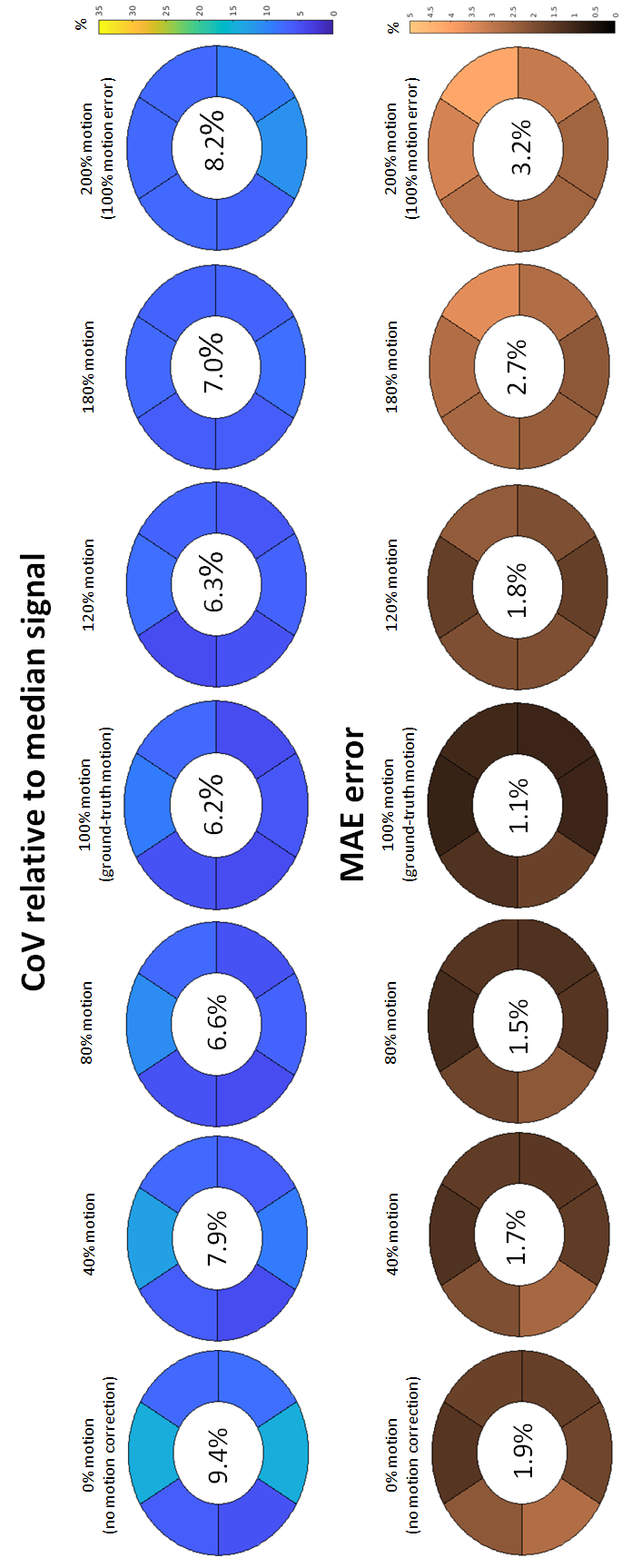
^

**Supporting Information Figure S4.** Coefficient of variation (CoV) and mean absolute error (MAE) for (unregularized) LRMC reconstructions considering a range of motion errors, from 0% (no motion) to 200% (i.e., 100% error with respect to ground-truth). CoV increase with motion errors (i.e. away from 100% motion), although all cases are substantially lower than zero-filing (Figure B). MAE present similar behaviour, although higher errors are observed for large positive motion errors (200%, i.e., 100% error with respect to ground-truth).

^
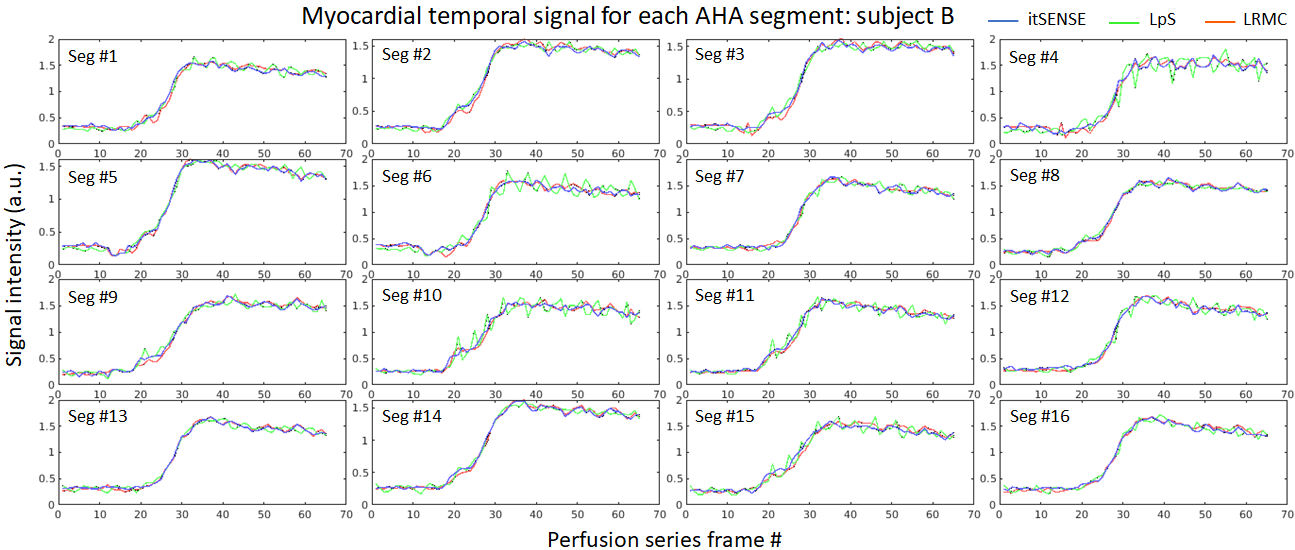

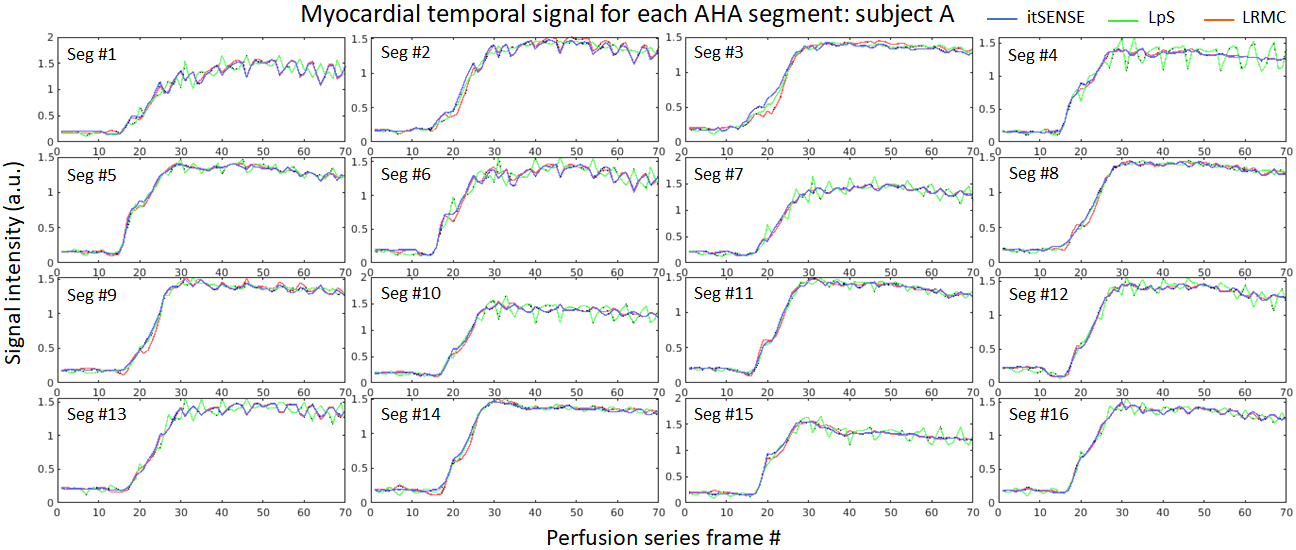
^

**Supporting Information Figure S6.** Signal evolutions of the perfusion series for subject B, along each of the 16 AHA myocardial segments, for iterative SENSE (blue), Low Rank plus Sparse (green) and the proposed Low Rank Motion Correction (red). itSENSE and LpS have been motion aligned using the same motion fields in LRMC to facilitate the comparison of the perfusion temporal evolutions. All three methods present similar temporal evolutions.

**Supporting Information Figure S5.** Signal evolutions of the perfusion series for subject A, along each of the 16 AHA myocardial segments, for iterative SENSE (blue), Low Rank plus Sparse (green) and the proposed Low Rank Motion Correction (red). itSENSE and LpS have been motion aligned using the same motion fields in LRMC to facilitate the comparison of the perfusion temporal evolutions. All three methods present similar temporal evolutions.

^
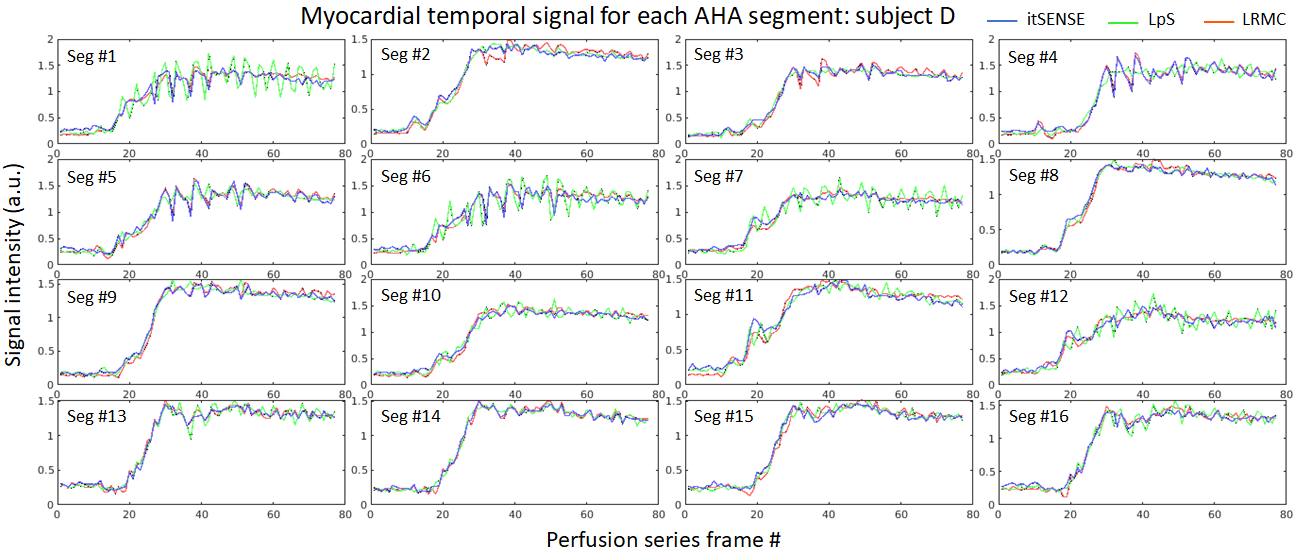

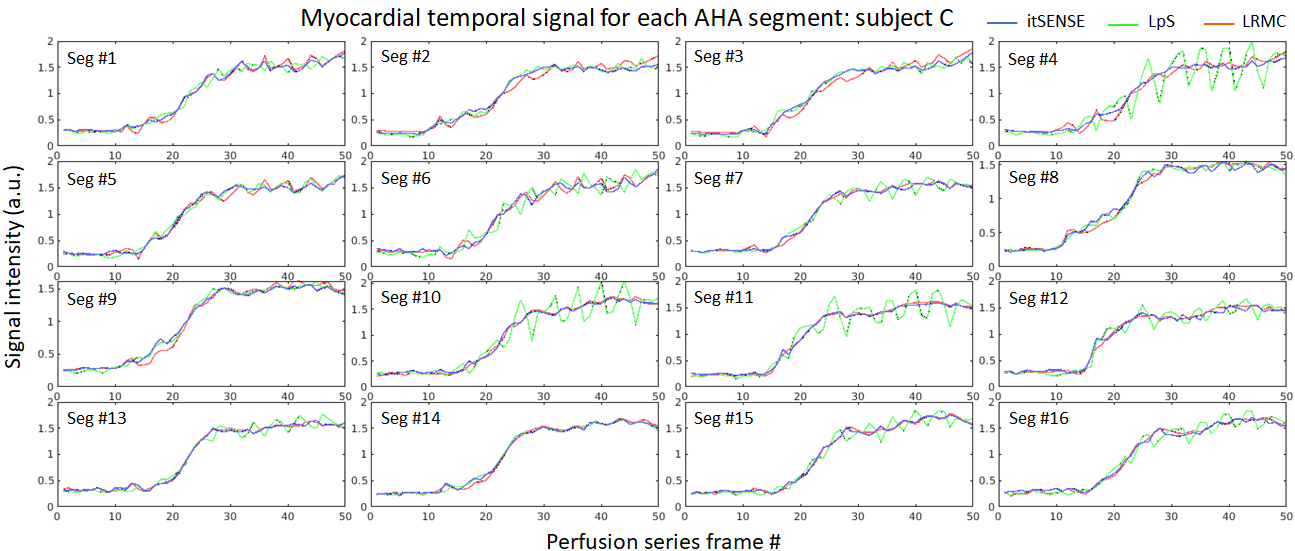
^

**Supporting Information Figure S8.** Signal evolutions of the perfusion series for subject D, along each of the 16 AHA myocardial segments, for iterative SENSE (blue), Low Rank plus Sparse (green) and the proposed Low Rank Motion Correction (red). itSENSE and LpS have been motion aligned using the same motion fields in LRMC to facilitate the comparison of the perfusion temporal evolutions. All three methods present similar temporal evolutions.

**Supporting Information Figure S7.** Signal evolutions of the perfusion series for subject C, along each of the 16 AHA myocardial segments, for iterative SENSE (blue), Low Rank plus Sparse (green) and the proposed Low Rank Motion Correction (red). itSENSE and LpS have been motion aligned using the same motion fields in LRMC to facilitate the comparison of the perfusion temporal evolutions. All three methods present similar temporal evolutions.

^
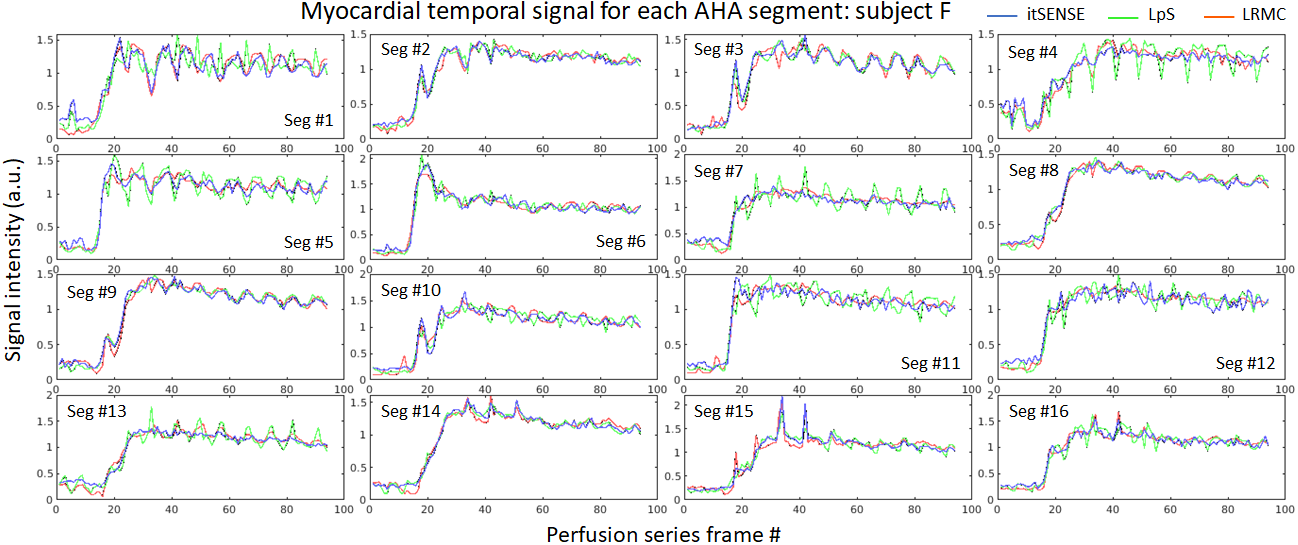

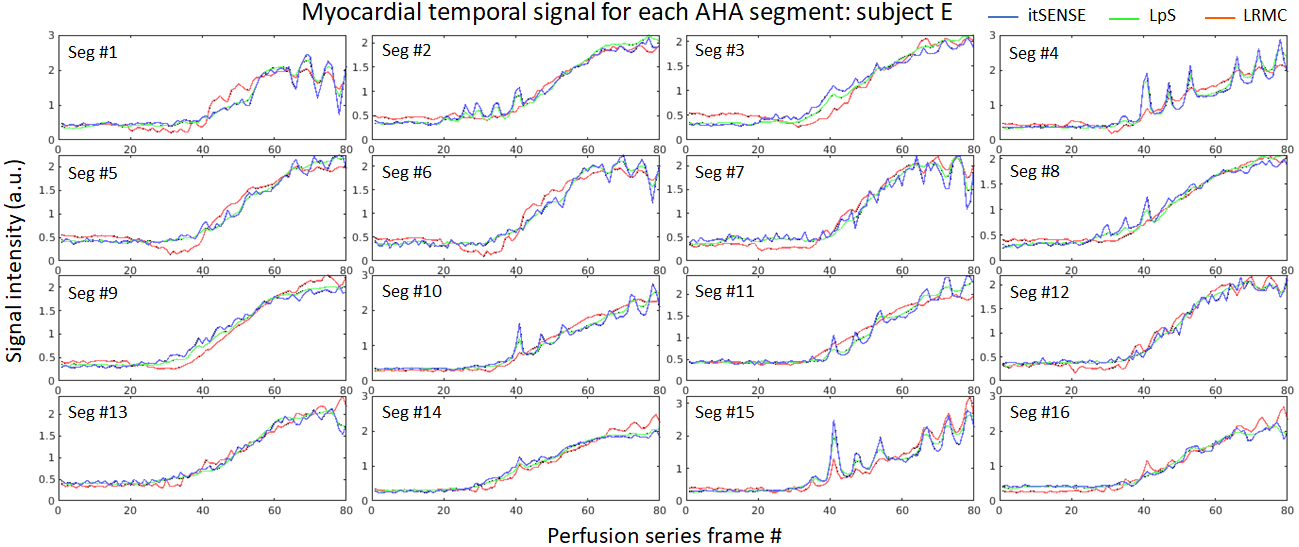
^

**Supporting Information Figure S10.** Signal evolutions of the perfusion series for subject F, along each of the 16 AHA myocardial segments, for iterative SENSE (blue), Low Rank plus Sparse (green) and the proposed Low Rank Motion Correction (red). itSENSE and LpS have been motion aligned using the same motion fields in LRMC to facilitate the comparison of the perfusion temporal evolutions. All three methods present similar temporal evolutions.

**Supporting Information Figure S9.** Signal evolutions of the perfusion series for subject E, along each of the 16 AHA myocardial segments, for iterative SENSE (blue), Low Rank plus Sparse (green) and the proposed Low Rank Motion Correction (red). itSENSE and LpS have been motion aligned using the same motion fields in LRMC to facilitate the comparison of the perfusion temporal evolutions. All three methods present similar temporal evolutions.

^
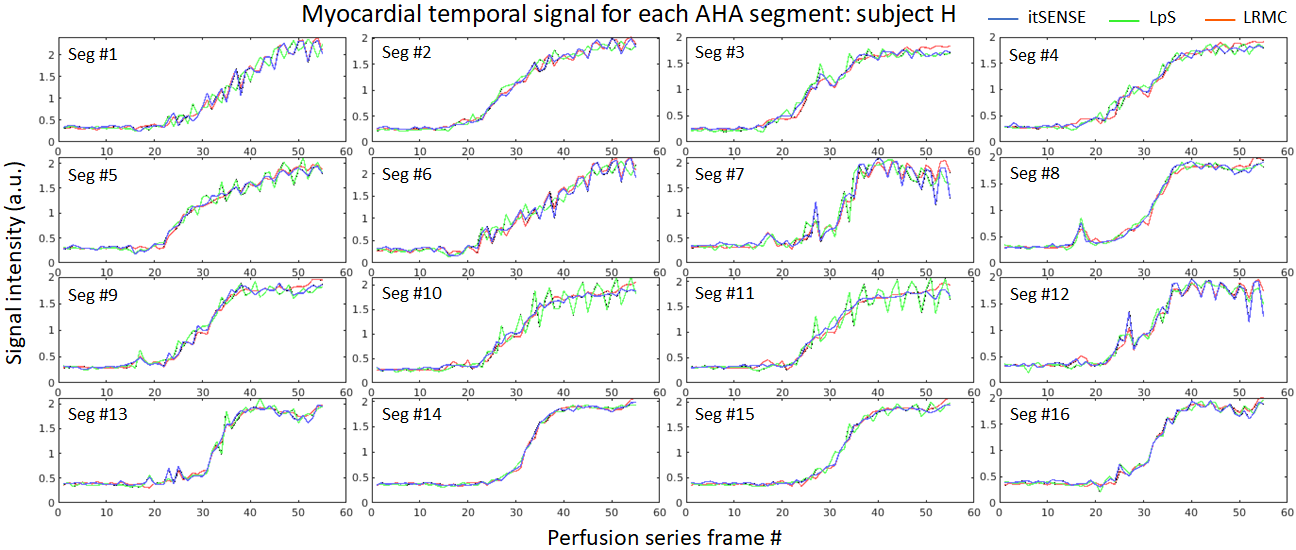

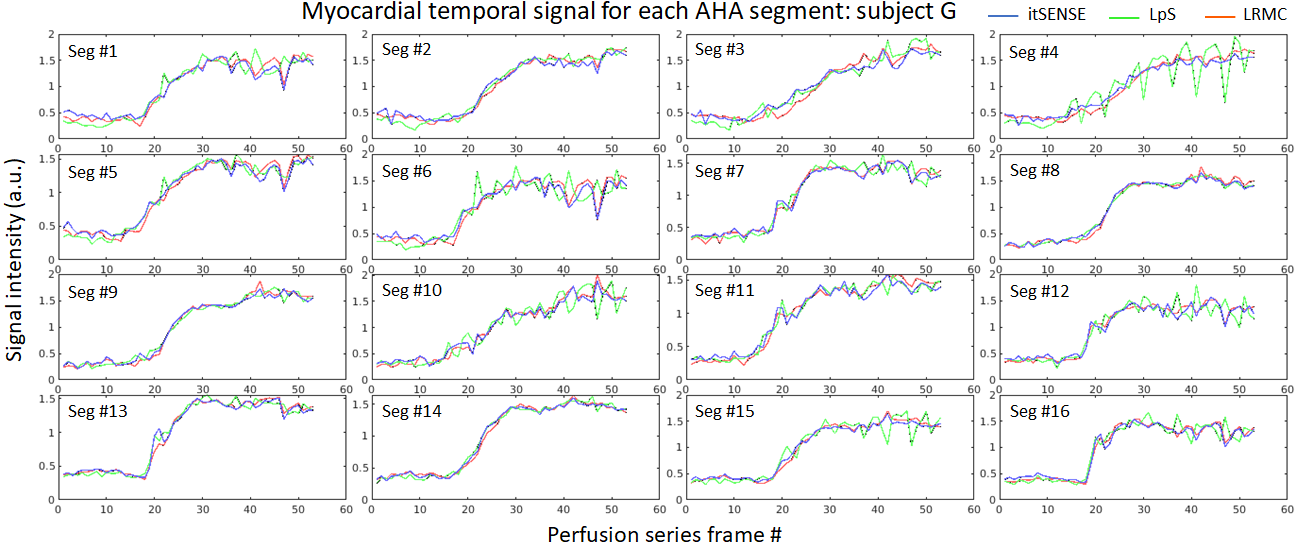
^

**Supporting Information Figure S12.** Signal evolutions of the perfusion series for subject H, along each of the 16 AHA myocardial segments, for iterative SENSE (blue), Low Rank plus Sparse (green) and the proposed Low Rank Motion Correction (red). itSENSE and LpS have been motion aligned using the same motion fields in LRMC to facilitate the comparison of the perfusion temporal evolutions. All three methods present similar temporal evolutions.

**Supporting Information Figure S11.** Signal evolutions of the perfusion series for subject G, along each of the 16 AHA myocardial segments, for iterative SENSE (blue), Low Rank plus Sparse (green) and the proposed Low Rank Motion Correction (red). itSENSE and LpS have been motion aligned using the same motion fields in LRMC to facilitate the comparison of the perfusion temporal evolutions. All three methods present similar temporal evolutions.

^
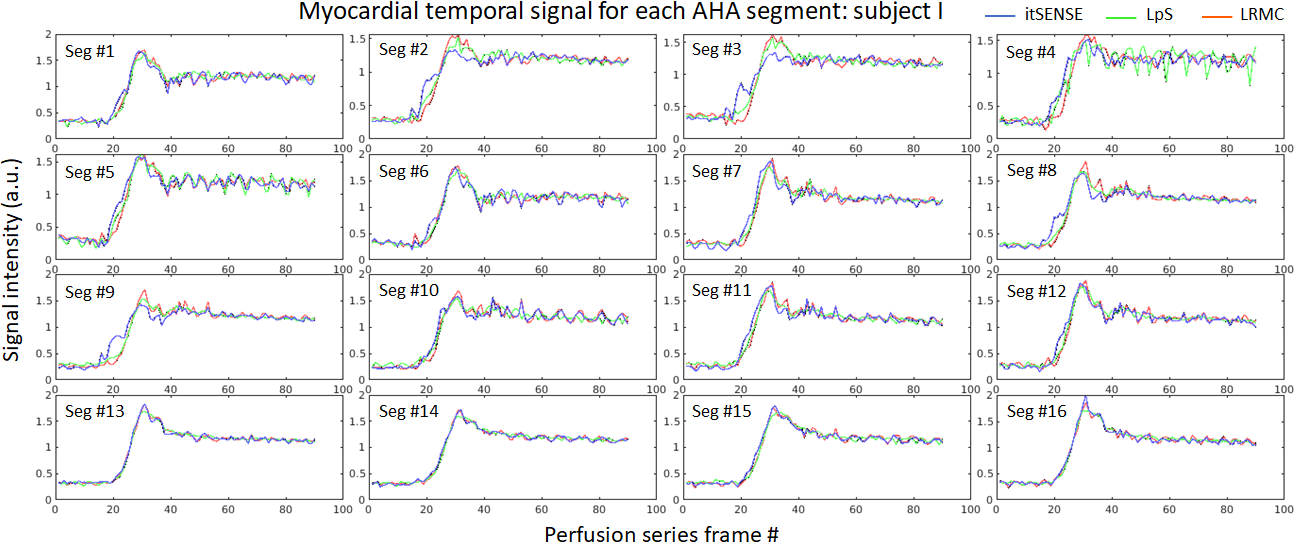
^

**Supporting Information Figure S13.** Signal evolutions of the perfusion series for subject I, along each of the 16 AHA myocardial segments, for iterative SENSE (blue), Low Rank plus Sparse (green) and the proposed Low Rank Motion Correction (red). itSENSE and LpS have been motion aligned using the same motion fields in LRMC to facilitate the comparison of the perfusion temporal evolutions. All three methods present similar temporal evolutions.

^
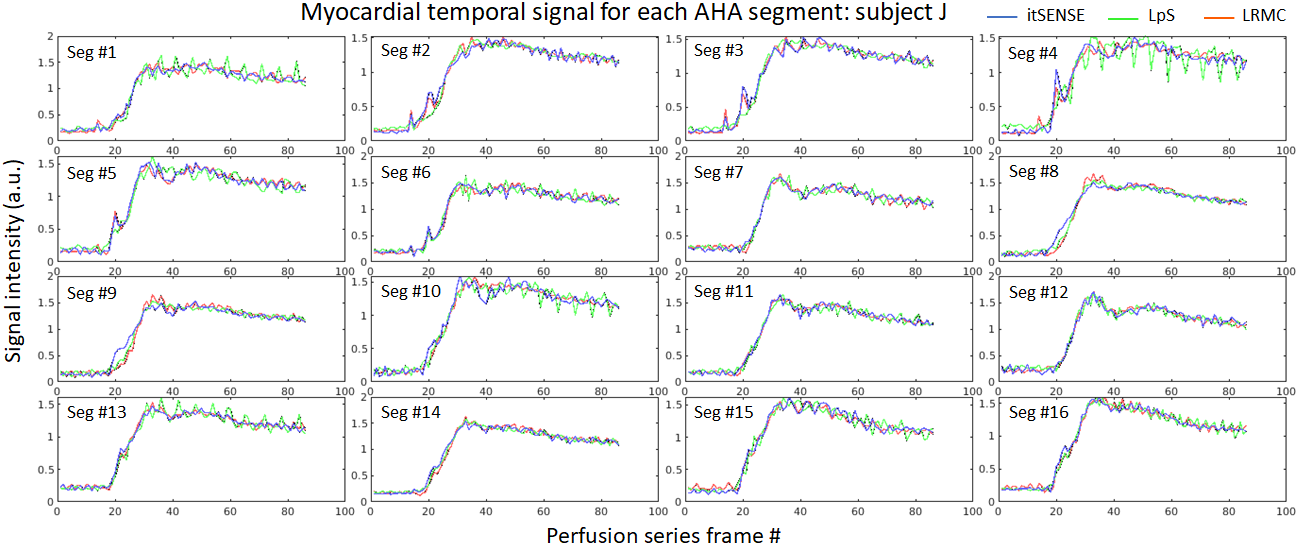
^

**Supporting Information Figure S14.** Signal evolutions of the perfusion series for subject J, along each of the 16 AHA myocardial segments, for iterative SENSE (blue), Low Rank plus Sparse (green) and the proposed Low Rank Motion Correction (red). itSENSE and LpS have been motion aligned using the same motion fields in LRMC to facilitate the comparison of the perfusion temporal evolutions. All three methods present similar temporal evolutions.
